# Head-to-Head Comparison of ^68^Ga-Prostate-Specific Membrane Antigen PET/CT and Ferumoxtran-10–Enhanced MRI for the Diagnosis of Lymph Node Metastases in Prostate Cancer Patients

**DOI:** 10.2967/jnumed.120.258541

**Published:** 2021-01-30

**Authors:** Melline G.M. Schilham, Patrik Zamecnik, Bastiaan M. Privé, Bas Israël, Mark Rijpkema, Tom Scheenen, Jelle O. Barentsz, James Nagarajah, Martin Gotthardt

**Affiliations:** 1 Department of Medical Imaging, Nuclear Medicine, Radboud University Medical Centre, Nijmegen, The Netherlands; and; 2 Department of Nuclear Medicine, Technical University Munich, Klinikum rechts der Isar, Munich, Germany

**Keywords:** prostate cancer, lymph node, prostate specific membrane antigen, 68Ga-PSMA PET/CT, ferumoxtran-10

## Abstract

Accurate assessment of lymph node (LN) metastases in prostate cancer (PCa) patients is critical for prognosis and patient management. Both prostate-specific membrane antigen (PSMA) PET/CT and ferumoxtran-10 nanoparticle–enhanced MRI (nano-MRI) are imaging modalities with high potential to identify LN metastases in PCa patients. The aim of this study was to compare the results of these imaging technologies in terms of characteristics and anatomic localization of suspicious LNs in order to assess the feasibility of their complementary use for imaging in PCa patients. **Methods:** In total, 45 patients with either primary PCa (*n* = 8) or recurrence (*n* = 36) were included in this retrospective study. All patients underwent both ^68^Ga-PSMA PET/CT and nano-MRI between October 2015 and July 2017 within 3 wk. Both scans were performed at the same institution according to local clinical protocols. All scans were analyzed independently by experienced nuclear medicine physicians and radiologists. The size, anatomic location, and level of suspicion were determined for all visible LNs. Subsequently, the findings from ^68^Ga-PSMA PET/CT and nano-MRI were compared without respect to a reference standard. **Results:** In total, 179 suspicious LNs were identified. Significantly more suspicious LNs per patient were detected by nano-MRI (*P* < 0.001): 160 were identified in 33 patients by nano-MRI, versus 71 in 25 patients by ^68^Ga-PSMA PET/CT. Of all suspicious LNs, 108 were identified only by nano-MRI (60%), 19 (11%) only by ^68^Ga-PSMA PET/CT, and 52 (29%) by both methods. The mean size of the suspicious LNs as identified by nano-MRI was significantly smaller (5.3 mm) than that by ^68^Ga-PSMA PET/CT (6.0 mm; *P* = 0.006). The median level of suspicion did not differ significantly. Both modalities identified suspicious LNs in all anatomic regions of the pelvis. **Conclusion:** Both modalities identified suspicious LNs that were missed by the other. Both modalities identified suspicious LNs in all anatomic regions of the pelvis; however, nano-MRI appeared to be superior in detecting smaller suspicious LNs. These findings suggest that nano-MRI has a potential role as a complement to PSMA PET/CT. However, since the clinical implications of the different results are not well established yet, further investigation of this complementary use is encouraged.

## Visual Abstract

**Figure:**
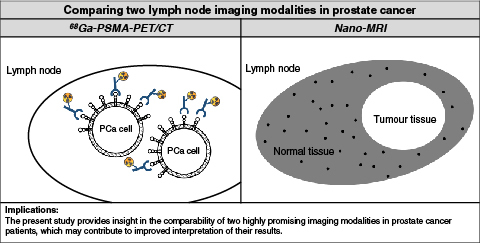

Detecting lymph node (LN) metastases in prostate cancer (PCa) patients is critical for prognosis and patient management. The current gold standard to assess the LN status is extended pelvic LN dissection (PLND). However, this procedure is invasive and associated with considerable morbidity (*1*). Previous research demonstrated that in a substantial number of patients (60%–85%), LN metastases were located outside the extended PLND template (*2–4*), illustrating the demand and increasing role for noninvasive imaging techniques to detect LN metastases in PCa patients.

Since conventional imaging techniques—that is, CT and MRI—use only morphologic criteria for LN assessment, and in PCa more than 60% of LN metastases are present in normal-sized LNs (<8 mm), these techniques are of limited value in LN staging (*5,6*), leading to the development of advanced functional and molecular imaging techniques. Recently, prostate-specific membrane antigen (PSMA)–based PET/CT was introduced. PSMA is a cell-surface glycoprotein that is overexpressed on more than 90% of PCa cells (*7*). Small molecules with high binding affinity to PSMA are labeled with positron emitters to enable whole-body tumor detection using PET/CT. Whereas data on accuracy were based predominantly on retrospective research (*8*), a large prospective study by Hofman et al. recently demonstrated sensitivity and specificity of 0.85 and 0.98, respectively, for both LN and distant metastases (*9*). The rapid implementation of this technique in several PCa guidelines affirmed the demand for accurate staging methods (*10,11*).

Another potential imaging modality for LN staging is MR lymphography or nanoparticle-enhanced MRI (nano-MRI). In nano-MRI, ultra-small superparamagnetic iron oxide particles (ferumoxtran-10 [Ferrotran; SPL Medical BV]) are used as a contrast agent. Through accumulation of these particles in normal lymphatic tissue after intravenous drip infusion, nano-MRI allows differentiation of metastatic LNs from benign LNs, irrespective of nodal size (*5,12*). The reported sensitivity and specificity in the detection of LN metastases in PCa patients are 82% and 93%, respectively (*12*). A metaanalysis reported sensitivities of up to 90% and specificities of up to 96% for various cancers, including PCa (*13*).

Published data suggest that PSMA PET/CT and nano-MRI are the imaging modalities with the highest reported accuracy to detect LN metastases (*9,14,15*). Since both modalities rely on different technical and biologic features, it was hypothesized that a combined use could even improve LN detection. Therefore, the goal of this study was to investigate the feasibility of a potential complementary role for these imaging modalities by comparing their results in the same patient and identifying differences and similarities in detected LN characteristics without respect to a reference standard.

## MATERIALS AND METHODS

### Patient Population

Forty-five patients were enrolled in this retrospective study. Before the database creation, the institutional review board approved this study and the requirement to obtain informed consent was waived (CMO2019.5810). The study included all patients with either primary PCa (*n* = 8) or recurrent disease (*n* = 36) who underwent both nano-MRI and ^68^Ga-PSMA PET/CT in our center between October 2015 and July 2017. The 2 scans needed to be performed within 3 wk of each other for inclusion. Patient characteristics were retrospectively collected from medical files.

### ^68^Ga-PSMA-HBED-CC PET/CT

^68^Ga-PSMA PET/CT was performed using an integrated PET/CT system (Biograph mCT 4-ring, 40-slice time-of-flight PET/CT scanner; Siemens Healthcare). For all patients, ^68^Ga-PSMA-HBED-CC was manufactured by the Radboud Translational Medicine Facility. The PET acquisition was 4 min per bed position for the pelvic area and 3 min for the rest of the body. A low-dose CT scan (slice thickness, 5.0 mm) was acquired for attenuation correction and image coregistration. PET/CT images were reconstructed in 3 orientations (axial, coronal, and sagittal). The administered dose of the tracer was 2 MBq/kg of body weight, and imaging was initiated after an approximately 60-min incubation time.

### Nano-MRI

All patients received ferumoxtran-10 intravenously in a weight-adapted dose of 2.6 mg/kg of body weight 24–36 h before the MRI scan. Ferumoxtran-10 was diluted in 100 mL of 0.9% NaCl solution and administered via drip infusion using a 0.22-μm-pore filter (Minisart NML syringe filter, catalog no. 16534-k; Sartorius AG). The infusion was performed at a slow rate of 1 mL/min at the start, increasing to 4 mL/min. The infusion duration was approximately 45 min and supervised by radiologists. MRI was performed using a 3-T MRI scanner (Magnetom Skyra or Trio; Siemens Healthineers). The imaging area included the pelvis from the pubic bone to the aortic bifurcation. The MRI protocol consisted of an isotropic 3-dimensional T1-weighted gradient-echo sequence (repetition time, 6.5 ms; echo time, 2.5 ms; flip angle, 10°; and spatial resolution, 0.9-mm isotropic) and an isotropic 3-dimensional iron-sensitive T2*-weighted gradient-echo sequence with fat saturation (multiple-echo data image combination, with repetition time, 21 ms; echo time, 12 ms; 3 combined echoes; flip angle, 10°; and spatial resolution, 0.85-mm isotropic).

### Image Analysis

All ^68^Ga-PSMA PET/CT exams were retrospectively reviewed by 2 certified nuclear physicians in consensus, and the nano-MR images were independently reviewed by 1 experienced radiologist. For both modalities, the number, anatomic location, and size of detected LNs were reported. The location was described according to preconfigured anatomic locations in the pelvis, consistent with clinical practice in our department. LN size was measured (mm) for the smallest axis. Additionally, all detectable LNs were classified with a level of suspicion (LoS) for both nano-MRI and ^68^Ga-PSMA PET/CT. This classification is a 5-point likeliness scale for potential malignancy that is used by nuclear physicians and radiologists in our center. For nano-MRI, LoS was based on the signal intensity in the iron-sensitive T2*-weighted MRI sequence and its distribution within the LN based on the diagnostic description proposed by Anzai et al. (*16*). LoS for ^68^Ga-PSMA PET/CT was based on the proposed criteria of the ^68^Ga-PSMA reporting and data system by Rowe et al. (*17*). This evaluation comprised a combination of tracer uptake, location, and size. In more detail, LNs with no tracer uptake were given an LoS of 1, defined as a high probability of being benign. LNs with equivocal tracer uptake at sites atypical of PCa involvement (e.g., axillary or hilar) were given a LoS of 2 (probably benign). A LoS of 3 (equivocal), was given to LNs with equivocal tracer uptake at sites typical of PCa involvement, LNs with intense uptake at sites highly atypical of PCa (i.e., the likelihood of nonprostatic malignancies or other [benign] origins is high), or LNs without tracer uptake but with pathologic aspects suspicious of malignancy on anatomic imaging. LNs with clearly increased tracer uptake at sites typical of PCa involvement but lacking definitive findings on anatomic imaging were given an LoS of 4, or probably malignant. A LoS of 5, defined as a high probability of being malignant, was given to LNs with intense tracer uptake at sites typical of PCa and with corresponding pathologic findings on anatomic imaging. For both modalities, LNs with a LoS of 3 or higher were considered suspicious and taken for statistical evaluation.

### Outcome Measurements and Statistical Analysis

Statistical analyses were performed using SPSS software, version 25. Descriptive statistical methods were used to characterize the patient cohort. For continuous data, mean and SD were reported. For categoric data, median and interquartile range were described. Only nonparametric statistical tests (Mann–Whitney *U* test and Wilcoxon signed-rank test) were performed since all data were nonnormally distributed. A *P* value of less than 0.05 was considered statistically significant.

## RESULTS

Forty-five patients underwent nano-MRI and ^68^Ga-PSMA PET/CT within a mean of 3 d (range, 1–18 d) between October 2015 and July 2017. The mean age of the patients was 64 y (range, 48–82 y). For the total cohort, the mean prostate-specific antigen (PSA) level at the time of scanning was 9.9 ng/mL (range, 0.1–150 ng/mL). For the subgroup of patients who underwent imaging for primary staging (*n* = 8), the mean PSA level was 28.9 ng/mL (range, 5.6–150 ng/mL). The mean PSA level in patients with recurrent disease (*n* = 33) was 5.0 ng/mL (range, 0.1–46 ng/mL). Detailed patient characteristics are described in Table 1. The median administered dose of ^68^Ga-PSMA-HBED-CC was 158 MBq (interquartile range, 133–180 MBq).

**TABLE 1:** Patient Characteristics

Characteristic	Data
Patients	45 (100%)
Age (y)	64 (48–82)
Serum PSA level (ng/mL)*	
Overall, *n* = 42	9.9 (0.0–150)
Primary setting, *n* = 8	28.9 (5.6–150)
Recurrence setting, *n* = 33	5.0 (0.0–46)
Time between diagnosis and scans (mo)^†^	50 (1–202)
Time between scans (d)	3.0 (1–18)
Before imaging	
Any PCa treatment	
Yes	36 (80%)
No	8 (18%)
Unknown	1 (2%)
PLND	
Yes	22 (49%)
No	19 (42%)
Unknown	4 (9%)
Clinical ISUP grade	
1	5 (11%)
2	6 (13%)
3	7 (16%)
4	13 (29%)
5	8 (18%)
Unknown	6 (13%)

* No data available for 3 patients.

† No data available for 1 patient.

ISUP = International Society of Urological Pathology.

Qualitative data are number and percentage; continuous data are mean and range.

**FIGURE 1.**
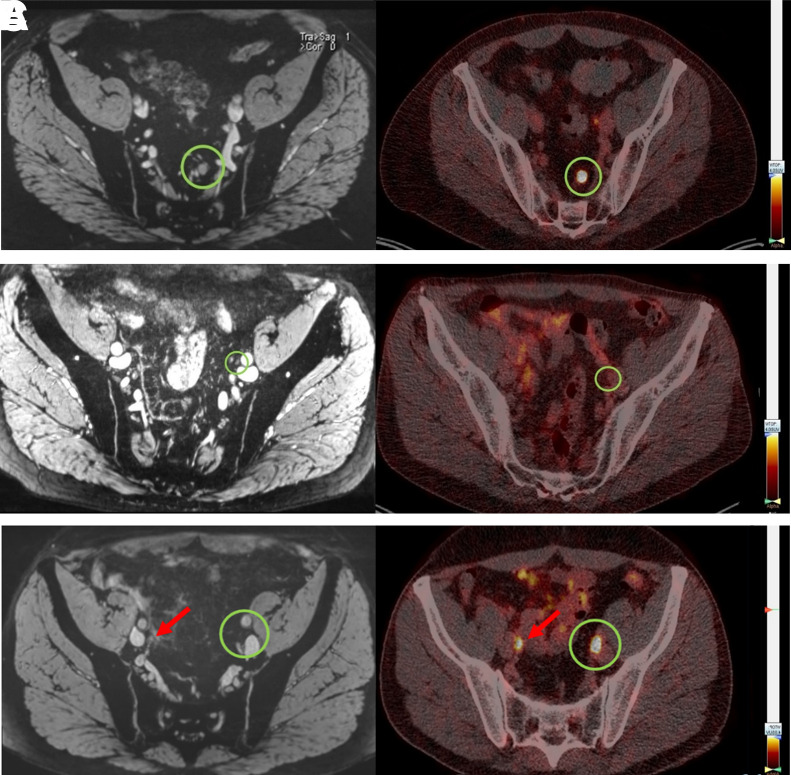
Examples of iron-sensitive T2*-weighted fat-saturated nano-MRI images (left) and PSMA PET/CT images (right). (A) Seven-millimeter-diameter LN in left pararectal region that is positive on both nano-MRI and PSMA PET/CT. (B) Four-millimeter-diameter LN in region of left external iliac artery that is positive on nano-MRI but negative on PSMA PET/CT. (C) Suspicious LN dorsal to left external iliac artery that is negative on nano-MRI (nano-MRI also shows no left ureter in this area) but positive on PSMA PET/CT. LNs are encircled; arrows indicate right ureter.

**FIGURE 2.**
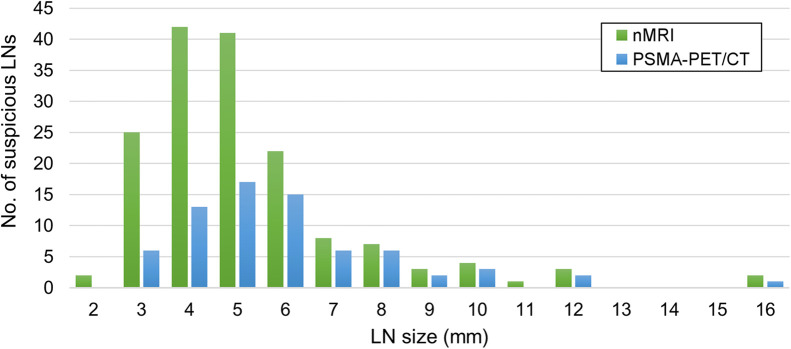
Size distribution of suspicious LNs as detected by nano-MRI (nMRI) and PSMA PET/CT.

A cumulative total of 179 suspected LNs (LoS ≥ 3) was identified in 33 patients. Examples of suspicious LNs as identified by nano-MRI, ^68^Ga-PSMA PET/CT, or both are shown in Figure 1. The characteristics of the nano-MRI and ^68^Ga-PSMA PET/CT results are shown in Table 2. In total, 179 suspicious LNs were identified. A significantly greater number of suspicious LNs were detected by nano-MRI (*P* < 0.001): 160 were identified in 33 patients by nano-MRI, versus 71 in 25 patients by ^68^Ga-PSMA PET/CT. Thus, per patient, nano-MRI identified a significantly greater number of suspicious LNs (mean, 3.6; range, 0–15) than ^68^Ga-PSMA PET/CT (mean, 1.6; range, 0–12) (*P* < 0.001). The difference in the size of the detected suspicious LNs between the 2 modalities is shown in Figure 2. The mean size of the suspicious LNs identified by nano-MRI (5.2 mm; range, 2–16) was significantly smaller than that identified by ^68^Ga-PSMA PET/CT (6.0 mm; range, 3–16 mm) (*P* = 0.006).

**TABLE 2:** Node Detection and Characteristics for Nano-MRI and PSMA PET/CT

Characteristic	Total	Nano-MRI	PSMA PET/CT	*P*
Total scans	90 (100%)	45 (100%)	45 (100%)	
Total positive scans	58 (64%)	33 (73%)	25 (56%)	
Total suspicious LNs	179 (100%)	160 (89%)	71 (40%)	
Suspicious LNs per patient	4.0 (range, 0–6)	3.6 (range, 0–15)	1.6 (range, 0–12)	<0.001*
Suspicious LN size (mm)	5.2 (range, 2–16)	5.3 (range, 2–16)	6.0 (range, 3–16)	0.006^†^
LoS	4 (IQR, 4–5)	4 (IQR, 4–5)	5 (IQR, 4–5)	

* Positive scan defined as at least 1 LN with LoS ≥ 3.

† Wilcoxon signed-rank test.

‡ Mann–Whitney *U* test.

IQR = interquartile range.

Qualitative data are number and percentage; continuous data are mean and range or IQR.

Table 3 shows which LNs were identified by both modalities and which by only one. Most of the suspicious LNs were identified by nano-MRI alone (*n* = 108, 60%). Almost a third (*n* = 52, 29%) were identified by both modalities, and 19 (11%) were identified by ^68^Ga-PSMA PET/CT alone. Not surprisingly, LNs identified by both modalities were larger (mean size, 6.5 mm; range, 4–16 mm) than those identified by either of the modalities alone. In line with this finding is the higher LoS of LNs identified by both modalities than of LNs identified by one of the techniques alone.

**TABLE 3:** Conformity of Nano-MRI and PSMA PET/CT

		Suspicious LNs as detected by…	
Characteristic	Both nano-MRI and PSMA PET/CT	Nano-MRI only	PSMA PET/CT only
No. of patients	20	30	14
Total suspicious LNs	52 (29%)	108 (60%)	19 (11%)
Suspicious LNs per patient	1.2 (range, 0–10)	2.4 (range, 0–8)	0.4 (range, 0–3)
LN size (mm)	6.5 (range, 4–16)	4.7 (range, 2–16)	4.4 (range, 3–8)
LoS	5 (IQR, 4–5)	4 (IQR, 4–5)	3 (IQR, 3–4)

IQR = interquartile range.

Qualitative data are number and percentage; continuous data are mean and range or IQR.

**FIGURE 3.**
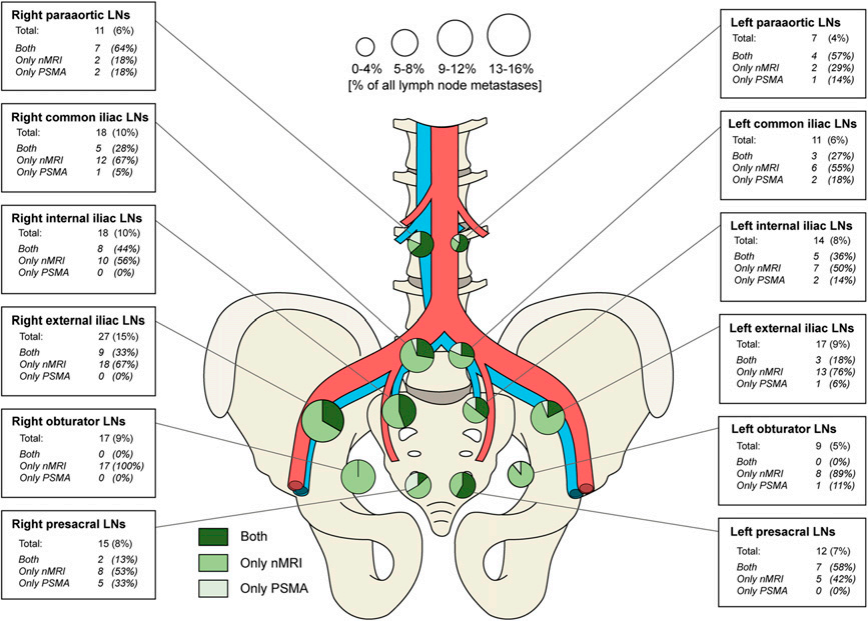
Anatomic distribution of identified suspicious LNs as detected by nano-MRI (nMRI) and PSMA PET/CT.

An overview of the anatomic localization of the suspicious LNs is depicted in Figure 3 (paraaortal and paravesical LNs are left out). Both modalities identified LNs across all anatomic locations, either left- or right-sided. Remarkably, 43% (*n* = 77) of all detected suspicious LNs were outside the standard extended PLND resection field (included in this field were the obturator, internal iliac, and external iliac regions).

## DISCUSSION

The aim of this study was to evaluate the feasibility of complementary use of 2 high-precision imaging techniques for the detection of metastatic LNs in PCa patients. We hypothesized that complementary use might even improve LN detection. Therefore, we aimed to identify differences in the number, size, LoS, and location of suspicious LNs in order to determine when this complementary role would be most pronounced. In this direct comparative study, 3 important results were achieved. First, nano-MRI identified a significantly greater number of suspicious LNs per patient than ^68^Ga-PSMA PET/CT (*P* < 0.001). Second, the LNs identified by nano-MRI were significantly smaller (*P* = 0.006) than those identified by ^68^Ga-PSMA PET/CT. Third, both modalities identified LNs throughout the pelvis in all anatomic regions, with, however, a significant number of suspicious LNs (43%) outside the standard extended PLND templates.

To the best of our knowledge, this was the first study directly comparing these specific imaging techniques. In 2005–2006, Fortuin et al. conducted comparable research comparing nano-MRI with ^11^C-choline-PET/CT. They showed that small suspicious LNs were detected at a higher rate by nano-MRI than by ^11^C-choline PET/CT (*18*), a finding consistent with our results. In recent years, however, MRI has continued to develop and improve, and new technologies (PSMA PET/CT) have emerged. To be more precise, technologic improvements compared with the MRI technique used by Fortuin et al. have led to an even higher spatial resolution (2 mm compared with 4 mm) in MRI. Additionally, PSMA-based PET/CT has already proven to be more sensitive than ^18^F-choline–based PET/CT (*19*). Consequently, reevaluation of these 2 imaging methods was considered valuable and led to the implementation of the current study.

The validation studies that have already been conducted for both imaging modalities showed promising sensitivity and specificity, and as technologic possibilities continue to evolve, accuracy is expected to improve further (*9,12*). Although there was no reference standard in this study, the main results provide insight into the complementary performance of the 2 modalities by identifying areas where they agree and disagree. Such results allow the definition of future areas of research that need to be addressed in order to define the optimal imaging strategy for PCa patients.

Our results show a potentially higher detection rate for nano-MRI than for ^68^Ga-PSMA PET/CT. As Figure 2 shows, disagreement was most pronounced on LNs smaller than 6 mm, suggesting size to be the most likely explanation for this difference. Recent research demonstrated large differences in the median histologic size of metastatic LNs that were detected—compared with those that were undetected—by ^68^Ga-PSMA PET/CT, suggesting a size-related sensitivity for LN metastasis detection by ^68^Ga-PSMA PET/CT (*20,21*). Possible explanations for this finding might be the biologic properties of PSMA expression on tumor tissue, as larger lesions are likely to have more PSMA receptors and thus higher tracer uptake. Yet, ^68^Ga-PSMA PET/CT was able to detect the smallest PSMA-positive lesions (below the spatial resolution of the scanner) when PSMA expression was highly concentrated but sometimes missed a larger lesion when PSMA expression was too dispersed (*20*). Furthermore, it has been demonstrated that PSMA expression correlates with International Society of Urological Pathology tumor grade and serum PSA level (*22,23*). Additionally, about 5%–10% of PCa lesions do not express PSMA (*7*). Since the iron-sensitive MRI sequence of nano-MRI has a higher spatial resolution (isotropic resolution of 0.85 mm) than PET/CT (6 mm), the resolution of nano-MRI enables detection of LNs down to a 2 mm^3^ voxel size. Thus, in contrast to nano-MRI, the performance of LN detection by PSMA PET/CT is largely dependent on tumor biology (*24*). Therefore, it could be anticipated that there is a potential advantage of nano-MRI in PCa patients with a lower International Society of Urological Pathology grade and PSA value. To draw solid conclusions from this disagreement on small suspicious LNs, more research is needed on the clinical significance of these small, potentially metastatic LNs and the biology of PSMA expression.

The difference in pathophysiologic targets between the 2 modalities (PSMA expression vs. lymphatic invasion of tumor tissue) could also partly explain our finding that nano-MRI identified suspicious LNs in 8 patients (8/45, 18%) who had no suspicious LNs on ^68^Ga-PSMA PET/CT. This finding suggests false-positive LNs for nano-MRI, a false-negative rate for ^68^Ga-PSMA PET/CT, or, most likely, a combination of both. In view of the different pathophysiologic targets, there are multiple explanations. Since about 5%–10% of tumor lesions do not show PSMA expression, these lesions will be missed by PSMA PET/CT (*7*). The fact that the sensitivity of ^68^Ga-PSMA PET/CT depends, in essence, on PSMA expression could explain the failure to detect lesions whose PSMA expression is too low. On the other hand, nano-MRI relies on the lymphotropic affinity to ferumoxtran-10 of macrophages, which accumulate the contrast agent in healthy LNs. Thus, when accumulation in nonmetastatic tissue is disturbed, such as by fibrosis, the discriminative ability between metastatic and nonmetastatic tissue in nano-MRI could be impaired. Ideally, a reference standard should be used to evaluate such results, and such research is strongly encouraged but surpassed the scope of the current study.

Contrary to the disagreement on size-related detection rates, another important finding was the agreement on anatomic localization; there were no anatomic regions in which either modality could not detect suspicious LNs (Fig. 3). In addition, both modalities identified a substantial number of suspicious LNs outside the extended PLND template (77/179, 43%). This finding was also described in recent research (*12,25,26*) and has a major impact on clinical care, as it challenges the diagnostic and therapeutic value of extended PLND (*27*), thus emphasizing the importance of accurate imaging modalities and explaining the current rapidly changing of clinical guidelines since the introduction of PSMA PET/CT (*9,28*).

This study was not without limitations. An important limitation of the study was its retrospective nature. Also, the studied population was relatively small and heterogeneous, as it consisted of both patients in the primary setting and patients in the biochemically recurrent setting. However, this limitation was due to the small number of patients who underwent both scans within a sufficiently tight time frame. Although the number of patients was limited, the population was unique and allowed us to compare the diagnostic performance of these imaging techniques without the disruptive effect of anatomic discordances. A final limitation was the lack of histopathologic confirmation of the identified suspicious LNs. Unfortunately, histologic or clinical confirmation of the positive LNs was impossible because most of our patient group was from abroad. Yet, the aim of this study was to compare the findings of both imaging modalities and discuss the potential clinical and scientific value (feasibility) of complementary use rather than to validate their findings.

## CONCLUSION

The findings of this comparison study imply potential benefit from complementary use of ^68^Ga-PSMA PET/CT and nano-MRI, most pronounced in small LNs. To make clinical recommendations for such complementary use, more profound prospective research on the competitive results is warranted and should focus on size-related issues and tumor biology (PSMA). Nevertheless, the results of this study underline the importance of understanding both the technical capabilities of imaging techniques and the tumor biology in order to interpret the imaging results appropriately.

## DISCLOSURE

Patrik Zamecnik is a scientific advisor to SPL Medical B.V. and has options in SPL Medical B.V. Jelle Barentsz is a scientific advisor to SPL Medical B.V. No other potential conflict of interest relevant to this article was reported.

**KEY POINTS**

**QUESTION:** How do the imaging results of ^68^Ga-PSMA PET/CT and nano-MRI compare in the same patients?
**PERTINENT FINDINGS:** In this retrospective head-to-head comparison study comprising 45 patients, nano-MRI identified a significantly greater number of suspicious LNs per patient (mean, 3.6) than did ^68^Ga-PSMA PET/CT (mean, 1.6), and the mean size of LNs detected by nano-MRI (mean, 5.3 mm) was significantly smaller than that detected by ^68^Ga-PSMA PET/CT (mean, 6.0 mm). Both modalities identified suspicious LNs in all anatomic pelvic regions.
**IMPLICATIONS FOR PATIENT CARE:** The present study of 2 highly promising imaging modalities in PCa patients provided insight into their comparability that may contribute to improved interpretation of results.
